# CRISPR-Cas12a-Assisted Genome Editing in *Amycolatopsis mediterranei*

**DOI:** 10.3389/fbioe.2020.00698

**Published:** 2020-06-26

**Authors:** Yajuan Zhou, Xinqiang Liu, Jiacheng Wu, Guoping Zhao, Jin Wang

**Affiliations:** ^1^CAS Key Laboratory of Synthetic Biology, Institute of Plant Physiology and Ecology, Shanghai Institutes for Biological Sciences, Chinese Academy of Sciences, Shanghai, China; ^2^University of Chinese Academy of Sciences, Beijing, China; ^3^School of Life Sciences and Technology, Shanghai Tech University, Shanghai, China; ^4^Department of Microbiology and Li Ka Shing Institute of Health Sciences, The Chinese University of Hong Kong, Prince of Wales Hospital, Shatin, Hong Kong; ^5^College of Life Sciences, Shanghai Normal University, Shanghai, China

**Keywords:** *Amycolatopsis mediterranei*, CRISPR-Cas12a, genome editing, NHEJ, HDR

## Abstract

*Amycolatopsis mediterranei* U32 is an industrial producer of rifamycin SV, whose derivatives have long been the first-line antimycobacterial drugs. In order to perform genetic modification in this important industrial strain, a lot of efforts have been made in the past decades and a homologous recombination-based method was successfully developed in our laboratory, which, however, requires the employment of an antibiotic resistance gene for positive selection and did not support convenient markerless gene deletion. Here in this study, the clustered regularly interspaced short palindromic repeat (CRISPR) system was employed to establish a genome editing system in *A. mediterranei* U32. Specifically, the *Francisella tularensis* subsp. *novicida Cas12a* (*FnCas12a*) gene was first integrated into the U32 genome to generate target-specific double-stranded DNA (dsDNA) breaks (DSBs) under the guidance of CRISPR RNAs (crRNAs). Then, the DSBs could be repaired by either the non-homologous DNA end-joining (NHEJ) system or the homology-directed repair (HDR) pathway, generating inaccurate or accurate mutations in target genes, respectively. Besides of *A. mediterranei*, the present work may also shed light on the development of CRISPR-assisted genome editing systems in other species of the *Amycolatopsis* genus.

## Introduction

*Amycolatopsis mediterranei* U32 is an industrial strain for production of rifamycin SV (Zhao et al., 2010), the first-line drug for anti-mycobacterial therapy till now (Rothstein, 2016). Due to the great importance of rifamycin, extensive efforts such as optimization of the fermentation conditions had been made to improve the yield of the antibiotics in the last century (Jiao et al., 1979; Lee et al., 1983; Mejia et al., 1998). Later, to facilitate the study of rifamycin biosynthesis as well as the molecular bioengineering of the producer, a genetic manipulation method based on native homologous recombination was developed for gene knockout in *A. mediterranei* (Ding et al., 2003). However, due to the relatively low efficiency of both DNA transformation and homologous recombination in *A. mediterranei*, an antibiotic cassette is usually employed to replace the target gene and the transformants are grown under antibiotic selection. To remove the antibiotic resistance cassette in the knockout mutant, the cassette should be flanked by site-specific recombination sequences such as the loxP sites or homologous arms, and a second cross-over recombination event is required. However, due to the relatively low genetic engineering efficiency, there are very few reports of successful construction of a markerless mutant in *A. mediterranei*. What is worse, since there are only a limited number of antibiotics applicable in *A. mediterranei*, it is difficult to perform continuous genetic engineering operations. Therefore, although the *Amycolatopsis* genus is well known to produce a huge diversity of secondary metabolites (Xu et al., 2014; Kumari et al., 2016; Adamek et al., 2018), the lack of efficient genome editing technology has severely impeded the research progress in this genus.

The clustered regularly interspaced short palindromic repeat (CRISPR) system is an adaptive immune system in bacteria and archaea (Horvath and Barrangou, 2010; Jinek et al., 2012; Mohanraju et al., 2016), where the CRISPR-associated (Cas) protein complex utilizes guide RNA for specific recognition, binding, and cutting of target nucleic acids with proper protospacer adjacent motifs (PAM) (Jinek et al., 2012). The CRISPR systems can be divided into class 1 and class 2 (Koonin et al., 2017), where the crRNA ribonucleoprotein (crRNP) effector of the class 1 system complexes are composed of multiple Cas proteins as subunits (Makarova et al., 2011, 2017a), whereas the class 2 system crRNP complexes contain single Cas protein such as the types II, V, and VI Cas proteins (Makarova et al., 2017b). With CRISPR-Cas-assisted accurate cleavage in target DNA sequences and thus introducing double-stranded DNA (dsDNA) breaks (DSBs), the genome engineering efficiency can be greatly improved. Up to date, both the type II Cas9 system and type V Cas12a system have been widely applied in genome editing in a large number of species (Cong et al., 2013; Jiang et al., 2014, 2015; Cobb et al., 2015; Huang et al., 2015; Matsu-Ura et al., 2015; Low et al., 2016; Jia et al., 2017; Harrison and Hart, 2018; Hu et al., 2019). Compared to Cas9, Cas12a has several distinct features, including the preference of T-rich PAM sequences and the staggered cleavage pattern against target dsDNA (Zetsche et al., 2015; Yamano et al., 2016). Besides, unlike Cas9, Cas12a only requires the CRISPR RNA (crRNA) but not the *trans*-activating RNA (tracrRNA), and is able to mature precursor crRNAs, thereby enabling Cas12a in multiple gene editing and regulation with much convenience (Fonfara et al., 2016; Zetsche et al., 2017).

Bacteria have evolved two mechanisms to efficiently repair DSB damage, including both homology-directed repairing (HDR) (Sung and Klein, 2006) and non-homologous DNA end-joining (NHEJ) (Lieber, 2010). Combined with the CRISPR system, HDR provides accurate and markerless target gene deletion, mutation, and insertion of foreign DNA sequences (Cobb et al., 2015). Alternatively, in some bacterial species such as *Mycobacterium smegmatis*, DSB can be repaired by the NHEJ system, which comprises an ATP-dependent DNA ligase and a Ku protein (Wright et al., 2017; Zheng et al., 2017). Unlike HDR, the NHEJ system does not require homologous DNA sequences for recombination, but directly joins the breaks, facilitating convenient gene deletion and insertion (Babynin, 2007). However, as the NHEJ repair may introduce errors at the joining site, it is inappropriate for accurate gene editing.

Here in this study, we successfully established the CRISPR-Cas12a-based genome editing system in *A. mediterranei* U32. We first demonstrated the existence of the NHEJ system in U32, and then combined NHEJ with Cas12a to construct site-specific markerless gene deletion mutants. Moreover, we also used the endogenous HDR to repair the Cas12a-introduced DSBs, facilitating efficient genome editing in U32.

## Methodology

### Strains, Media, and Growth Conditions

Strains and plasmids used in this study are listed in Supplementary Table S1. *Escherichia coli* DH10B was used for DNA cloning and was grown at 37°C in LB medium. *A. mediterranei* U32 was grown at 30°C in Bennet medium (yeast extract, 1 g/l; glycerol, 10 g/l; glucose, 10 g/l; beef powder, 1 g/l; tryptone, 2 g/l; and agar, 15 g/l; pH 7.0). To prepare the competent cells, *A. mediterranei* strains were cultured at 30°C in MYM medium (yeast extract, 3 g/l; malt extract, 3 g/l; peptone, 5 g/l; glucose, 10 g/l; sucrose, 170 g/l; KNO_3_, 6 g/l; glycine, 10 g/l; CaCl_2_⋅2H_2_O, 0.735 g/l; MgCl_2_⋅6H_2_O, 1.015 g/l; pH 7.0). For analyses of the growth phenotypes, *A. mediterranei* strains were grown in minimal medium (4% glucose, 0.05% NaCl, 0.2% K_2_HPO_4_, 0.1% MgSO_4_, 0.001% FeSO_4_⋅7H_2_O, 0.0001% MnCl_2_⋅4H_2_O, and 0.001% ZnSO_4_⋅7H_2_O) supplemented with either (NH_4_)_2_SO_4_ or KNO_3_ as the sole nitrogen sources. When necessary, appropriate antibiotics were added in the medium mentioned above at the following concentrations: 100 μg/ml ampicillin, 50 μg/ml kanamycin, 34 μg/ml chloramphenicol, 50 μg/ml apramycin, and 100 μg/ml hygromycin.

### Construction of CRISPR-Cas12a-Based Genome Editing Plasmids

Primers for plasmid construction, mutant verification, and Sanger sequencing are listed in Supplementary Table S2. The *Francisella tularensis* subsp. *novicida Cas12a* gene (*FnCas12a*, previously known as *FnCpf1*) was PCR amplified from the plasmid pJV53-Cpf1 (Yan et al., 2017) using primer of *FnCas12a*-F and *FnCas12a*-R. Then, the linearized vector was obtained through PCR amplification of pDZL803 (Li et al., 2017b) with primers of pDZL803-apr-F and pDZL803-apr-R, which contained the promoter region of the apramycin resistance gene (P_apr_). *Cas12a* gene and the linearized pDZL803 vector were then assembled using the Ezmax seamless assembly kit (Tolo Biotech., Shanghai, China), and the obtained recombinant plasmid pDZLCas12a was further confirmed by Sanger sequencing (Supplementary Figure S1).

The crRNA guide sequences are listed in Supplementary Table S3. First, the BpmI and HindIII restriction sites in pCR-Hyg were replaced by the BbsI and AseI sites, obtaining the plasmid pCR1. In detail, the plasmid pCR-Hyg (Yan et al., 2017) was employed as the template for PCR amplification with primers of pCR-HYG-F and pCR-HYG-R, followed by template removal with DpnI and then self-assembly with the Ezmax seamless assembly kit (Tolo Biotech.). Then, the crRNA expressing cassette, containing the hsp60 promoter, two crRNA Direct Repeats (DR) sequences, the BbsI and AseI sites for insertion of crRNA guide sequences and the *rrnB* T1 terminator, was further amplified from pCR1 with paired primers of hsp60-rrnB-F and hsp60-rrnB-R, and the amplicon was then inserted into plasmid pULVK2A (Kumar et al., 1994), generating plasmid pULcrRNA (Supplementary Figure S1). Plasmid pULVK2A was generated from pRL1 (Lal et al., 1991) by spontaneous deletion of DNA sequences during passage, and can stably self-replicate in *A. mediterranei*. The Cas12a-expressing plasmid pDZLCas12a and the crRNA-expressing plasmid pULcrRNA were used to test the effectiveness of the CRISPR/Cas12a system in U32.

### Deletion of *rifZ* and *glnR* Genes in U32

The CRISPR/Cas12a-assisted genome editing plasmids were constructed on the basis of plasmid pULcrRNA. First, 20-nt crRNA guide sequences for targeting *rifZ* and *glnR* were designed, synthesized, and individually annealed, and were then inserted into pULcrRNA that was digested by BbsI and AseI, generating pULrifZ and pULglnR, respectively. Then, 1.5-kb upstream and downstream sequences of the target genes (e.g., *rifZ* and *glnR*) were PCR amplified from U32 genome with paired primers (rifZL-F/rifZL-R, rifZR-F/rifZR-R, glnRL-F/glnRL-R, and glnRR-F/glnRR-R), the amplicons of which were used as homologous arms for HDR. The apramycin resistance gene was released from pBCAm plasmid by PstI digestion. Then, the upstream and downstream arms as well as the apramycin resistance cassette were assembled (designated as LAR donor fragment) by Ezmax seamless assembly kit (Tolo Biotech.) and introduced into the NdeI-treated plasmids of pULrifZ and pULglnR, obtaining the knout-out plasmids of pULrifZ-LAR and pULglnR-LAR, respectively (Supplementary Figures S1, S3). Alternatively, plasmids for markerless deletion of target genes were constructed through direct assembly of the upstream and downstream homologous arms and the crRNA expression cassette for guiding target-specific cleavage, and the obtained plasmids for *rifZ* and *glnR* markerless deletion were named pULrifZ-LR and pULglnR-LR, respectively (Supplementary Figure S1).

The U32 competent cells for electroporation were prepared as previously described (Ding et al., 2003). The Cas12a expression vector (pDZLCas12a) was electroporated into U32 competent cells and transformants were cultured on selective plates containing hygromycin. Specifically, about 500-ng pDZLCas12a was electroporated into 75-μl U32 competent cells with the following electroporation parameters: 1760 V, 1000 Ω, 25 μF, and 2 mm cuvette. Transformants were cultivated at 30°C for 7 days, and the clones were counted, analyzed, and verified by both PCR amplification and subsequent Sanger sequencing. The transformant expressing Cas12a was then employed for preparation of competent cells for subsequent gene editing. To test the NHEJ activities in U32, 300-ng crRNA-expressing plasmids of pULrifZ and pULglnR, targeting *rifZ* and *glnR*, respectively, were electroporated into the competent cells expressing Cas12a, and the transformants were then cultured and analyzed. Noticeably, there were no donor arms on plasmids pULrifZ and pULglnR for homologous recombination.

Similarly, to precisely delete target genes via HDR-mediated repair of DSBs, 300-ng plasmids of pULrifZ-LAR, pULrifZ-LR, pULglnR-LAR, and pULglnR-LR were individually electroporated into the Cas12a-expressing competent cells to delete the target gene *rifZ* and *glnR*, respectively. The transformants were cultivated on Bennet plate supplemented with apramycin at 30°C for 7 days, and the colonies were confirmed by colony PCR and Sanger sequencing.

### Phenotypic Analyses of Gene Deletion Mutants

To analyze the growth phenotypes of the *glnR* deletion mutants, mutants were cultured with minimal medium with 20 mM KNO_3_ or 10 mM (NH4)_2_SO_4_ as the sole nitrogen sources. Specifically, mutants were first grown in Bennet medium and the cells were then washed with nitrogen-free medium. After that, a 10-fold serial dilution was made from the starting OD_600_ density of 1–1/400, and diluted cells were then spotted onto minimal medium plate (Dadura et al., 2017) before being further incubated at 30°C for 5 days. Three independent experiments were performed.

## Results

As *A. mediterranei* is an important industrial strain for rifamycin production, many efforts have been made to study its genetic operation system, including the characterization of endogenous plasmids. However, up to now, there is only one stable replicon (namely, the pA-rep) characterized from the endogenous plasmid pA387 in *Amycolatopsis* sp. DSM 43387, and all self-replicable plasmids (e.g., pRL1 and pULVK2A) in *A. mediterranei* are generated from this plasmid (Lal et al., 1991; Kumar et al., 1994). Due to the plasmid incompatibility, it is hard to stably transform two plasmids with the same replication origin inside one cell. Therefore, to develop a CRISPR-based genetic engineering system in *A. mediterranei* U32, we decided to clone the *Cas* gene in an integrative plasmid and the crRNA expression cassette in a self-replicable plasmid. We ever tested the dead *SpCas9* gene from *Streptococcus pyogenes* (Jinek et al., 2012), and cloned it into an integrative plasmid, which was then electroporated into U32 competent cells. However, no transformants were obtained (data not shown) after repeated electroporation experiments, which indicated that the expression of *dCas9* alone was toxic to U32.

**FIGURE 1:**
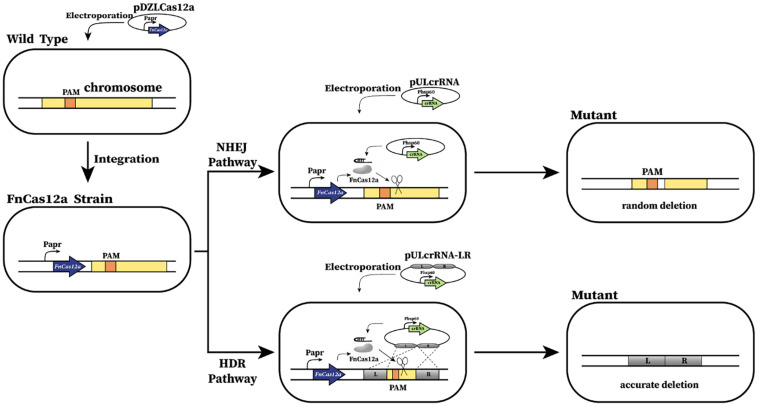
Schematic of CRISPR-Cas12a-assisted genome editing in *A. mediterranei* U32. First, *FnCas12a* was integrated into U32 chromosome and constitutively expressed under the promoter of P_apr_. Then, the crRNA transcribing plasmid was electroporated into the U32 competent cells that harbored FnCas12a. Guided by a target-specific crRNA, Cas12a specifically cleaved target dsDNA and generated DSBs on the chromosome, which could be repaired by either NHEJ or HDR, generating desired mutants. When DSBs were repaired by NHEJ, the breaks were directly jointed with no homologous donor DNA required; however, DNA sequences of ranging length around the Cas12a cleavage site will be deleted during the repair.

Then, instead of testing the wild-type Cas9, we tested the FnCas12a from *F. tularensis* (Zetsche et al., 2015), an alternative to Cas9 for CRISPR-mediated genome editing and has been successfully used in *M. smegmatis* (Yan et al., 2017). Similarly, the codon optimized *FnCas12a* gene was cloned in an integrative plasmid and was further electroporated into U32 competent cells to allow for integration into the *attB* site in the genome (Figure 1). Transformants were successfully obtained and the integrated *FnCas12a* gene was further confirmed by colony PCR verification and subsequent Sanger DNA sequencing; however, the transformation efficiency was much lower than that of the control plasmid with no *Cas12a* gene. Further phenotypic analysis showed that both bacterial growth and the rifamycin production of the transformant expressing FnCas12a were similar to those of the wild type U32 (Supplementary Figure S2), which implied that the constructed strain was a qualified system for genome editing analysis.

**FIGURE 2:**
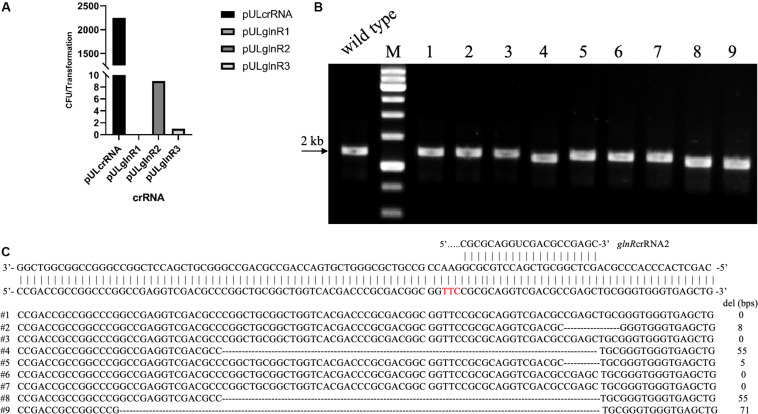
CRISPR-Cas12a-assisted *glnR* deletion in combination with the NHEJ repair in *A. mediterranei* U32. **(A)** The number of transformants obtained with different crRNAs transformed. Plasmid pULcrRNA, which contained no crRNA guide sequences, was employed as a control plasmid. The number stood for the total colonies obtained from three transformation experiments, where no transformants were obtained with crRNA1. The guide sequences are listed in Supplementary Table S3. **(B)** Characterization of pULglnR2 transformants by colony PCR amplification. M, GeneRuler 1 kb DNA Ladder (Thermo Scientific). Lanes 1–9, nine colonies transformed with CRISPR-Cas12a and pULglnR2 targeting *glnR* gene. **(C)** Sanger sequencing results of the PCR amplicons of the nine colonies in Figure 2B. Five colonies contained random deletion at the target site within *glnR* gene, which was repaired by the NHEJ pathway, and the deleted DNA sequences as well as the length were indicated. The PAM sequence was highlighted in red.

To test the whether Cas12a could introduce site-specific DSBs in U32, the *glnR* gene, which encodes the global regulator for nitrogen metabolisms, was chosen as the target gene. We designed three crRNAs targeting different coding regions in *glnR*, where crRNA1 and crRNA2 targeted the non-template strand (NT) and the crRNA-3 targeted the template strand (T) (Supplementary Table S3). The above three crRNAs were individually cloned into self-replicable pULVK2A, and the obtained plasmids were then electroporated into the U32 competent cells that constitutively expressed FnCas12a. In comparison with thousands of colonies obtained with the transformation of the control plasmid with no crRNA expression cassette, only 0, 9, and 1 transformants were obtained for plasmids pULglnR1, pULglnR2, and pULglnR3, respectively (Figure 2A), suggesting that the CRISPR-Cas12a system could efficiently cleave the genomic DNA.

On the other hand, although no homologous DNA sequences were introduced for homologous recombination, we still obtained some transformants (Figure 2A), and the nine transformants with pULglnR2 were further verified by PCR amplification of the target regions. The PCR results showed that the amplicons were of different sizes (Figure 2B), which indicated that there might be random deletions inside the target gene. We further confirmed this hypothesis by Sanger sequencing of the PCR amplicons and found five of nine transformants contained deletions (ranging from 5 to 71 bps) at the glnRcrRNA2 targeting site (Figure 2C). With the identification of conserved homologues of ATP-dependent DNA ligase and Ku protein (Supplementary Tables S4, S5), one may conclude that the DSBs were probably repaired by NHEJ in U32, although the possibility of other template-independent repair such as alternative end-joining (A-EJ) (Chayot et al., 2010) and microhomology-mediated end-joining (MMEJ) (Sfeir and Symington, 2015) cannot be completely excluded. Meanwhile, there might also exist unknown mechanisms to inactivate the CRISPR-Cas12a system in U32 as no mutations were found in the target region among the rest four clones. Collectively, above findings not only demonstrated that Cas12a generated site-specific DSBs but also suggested that there was NHEJ in U32. As a consequence, with the combination of CRISPR-Cas12a-induced site-specific DSBs and NHEJ-mediated repair, markerless gene mutations can be easily acquired in this bacterium.

**FIGURE 3:**
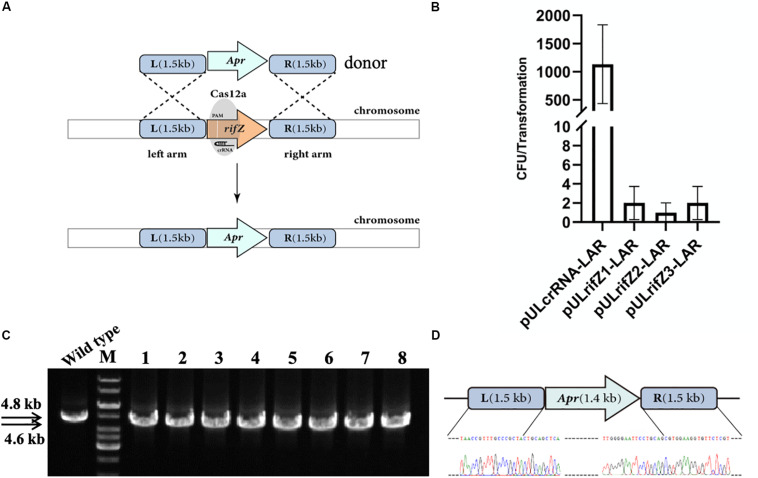
Replacement of *rifZ* by an antibiotic cassette with CRISPR-Cas12a-assisted precise cutting and HDR-mediated precise recombination in *A. mediterranei* U32. **(A)** Schematic of *rifZ* deletion. With the existence of a crRNA targeting *rifZ*, Cas12a introduced DSBs within *rifZ* gene, which could then be precisely repaired by a donor DNA fragment containing an apramycin resistance cassette and the flanking upstream and downstream homologous sequences. **(B)** The number of transformants obtained with three different crRNAs targeting different loci within *rifZ*. Plasmid pULcrRNA, which contained no crRNA guide sequences, was employed as a control plasmid. The data were obtained from three independent transformation experiments. **(C)** PCR amplification analysis of *rifZ* mutant with paired primers of *rifZ*-KO-F and *rifZ*-KO-R. The amplicon of the wild type was 4.8 kb in length, while was smaller in *rifZ* mutants. M, GeneRuler 1 kb DNA Ladder (Thermo Scientific); lanes 1 and 2, colonies obtained with pUL*rifZ*1-LAR; lanes 3 and 4, colonies obtained with pUL*rifZ*2-LAR; lanes 5–8, colonies obtained with pUL*rifZ*3-LAR. **(D)** Sanger sequencing results of the amplicon from Figure 3C. All eight amplicons were sequenced and found to be correct, and only the results of the first colony were presented.

Besides, we also combined the CRISPR-Cas12a-assisted target cleavage with endogenous HDR activity to precisely delete target genes. To measure the efficiency of the HDR-mediated precise genome editing, we next knocked out the *rifZ* gene encoding the rifamycin pathway-specific activator by replacing it with the apramycin resistance cassette (Figure 3A). Three crRNAs were designed to target both the T strand (rifZcrRNA-1 and rifZcrRNA-2) and the NT strand (rifZcrRNA-3) of *rifZ* (Supplementary Table S3). The resistance cassette was in fusion assembled with both upstream and downstream homologous arms of *rifZ*, and the obtained donor fragment was then introduced into the plasmids expressing *rifZ* targeting crRNAs, generating plasmids pULrifZ1-LAR, pULrifZ2-LAR, and pULrifZ3-LAR, respectively. The obtained three plasmids as well as a control plasmid were then individually electroporated into the U32 competent cells harboring *FnCas12a*. In comparison with the more than 1000 colonies obtained from the transformation of the control plasmid, less than 10 colonies on average were obtained with the three plasmids with *rifZ*-specific crRNAs (Figure 3B). Subsequently, eight colonies from the transformation of pULrifZ1-LAR were verified by both PCR amplification and Sanger sequencing, and the results unambiguously showed that the *rifZ* gene was precisely replaced with the apramycin resistance cassette in all tested colonies (Figures 3C,D).

**FIGURE 4:**
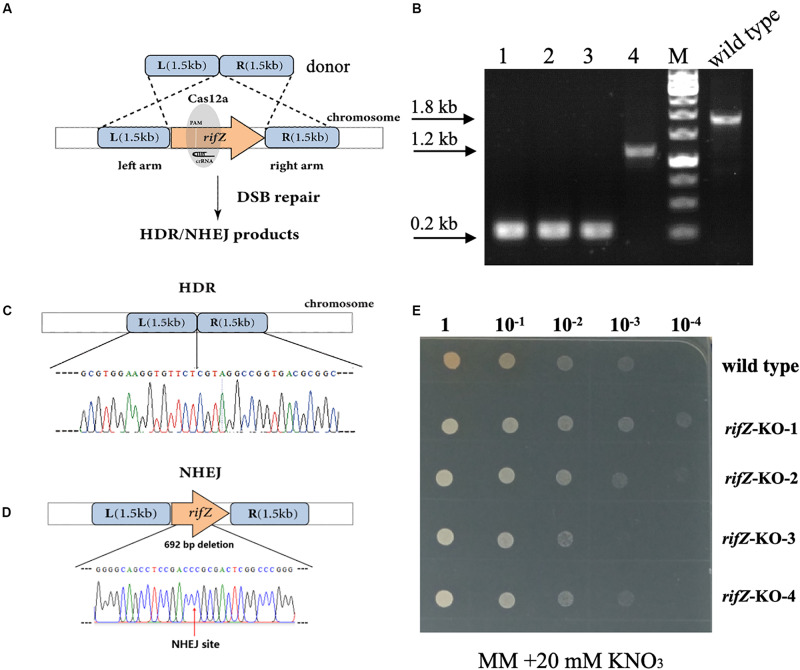
Markerless deletion of *rifZ* gene with the Cas12a and HDR systems in U32. **(A)** Schematic of markerless deletion of *rifZ* gene. Cas12a-generated DSBs in *rifZ* could be repaired by HDR. **(B)** Verification of *rifZ* deletion mutants by colony PCR with paired primers of rifZ+P-F and rifZ+P-R. M, GeneRuler 1 kb DNA Ladder (Thermo Scientific). Lanes 1–4 represented *rifZ*-KO-1 to *rifZ*-KO-4, respectively. **(C)** Sanger sequencing results from the amplicon of *rifZ*-KO-1 in Figure 4B. **(D)** Schematic of the *rifZ*-KO-4 mutant in Figure 4B. The mutant was obtained via NHEJ-mediated repair of the Cas12a-generated DSBs in *rifZ* gene. Confirmed by Sanger sequencing, 692-bp sequences were found to be deleted at the Cas12a cleavage site. **(E)** The growth phenotype of *rifZ* mutants on minimal medium. Serially diluted liquid culture was spotted on plates and cultured at 30°C for 5 days, and only the wild type produced pigmented rifamycin SV.

After confirmation of the effectiveness of HDR-mediated repair of CRISPR-Cas12a-generated DSBs, we then attempted to combine the CRISPR-Cas12a system and the endogenous HDR pathway to construct precise markerless mutants of both *rifZ* and *glnR*. The upstream and downstream homologous arms of the target genes were in fusion assembled and then inserted into the crRNA expressing plasmid, followed by electroporation into the U32 competent cells that constitutively expressed Cas12a protein (Figure 4A). For both target genes, i.e., *glnR* and *rifZ*, a dozen transformants were successfully obtained, which were further verified by both PCR amplification and Sanger sequencing. Among the four tested *rifZ* mutants, three had the HDR-assisted accurate *rifZ* gene deletion and one had inaccurate 692-bp deletion within *rifZ* gene, which was obviously repaired by NHEJ (Figures 4B–D). Phenotypic analysis showed that all four *rifZ* mutants produced no golden pigment (Figure 4E and Supplementary Figure S4a) and much reduced rifamycin SV yield (Supplementary Figure S4b) as indicated by the bactericidal test, which were consistent with the previous findings that RifZ functions as the pathway-specific activator for the whole *rif* cluster (Li et al., 2017a). Similarly, both the colony PCR and Sanger sequencing results demonstrated that the *glnR* gene, which encodes the central governor for nitrogen metabolisms, was precisely and markerlessly deleted (Supplementary Figures S5a,b) in all four tested transformants. Subsequent growth phenotypic analysis showed that all these *glnR* mutants grew poorly on minimal medium with nitrate as the sole nitrogen source (Supplementary Figure S5c). Collectively, above results clearly demonstrated that endogenous HDR pathway can be employed to efficiently repair the CRISPR-Cas12a-generated DSBs and engineer precise and markerless mutants.

## Discussion

In this study, we successfully employed CRISPR-Cas12a system to develop a genome editing system in *A. mediterranei*. To test the effectiveness of Cas12a-mediated site-specific DSBs, Cas12a and crRNAs were co-expressed in U32. And to our great surprise, even no homologous recombination arms were introduced, several transformants were obtained, leading to the identification of the endogenous NHEJ activities. Then, we showed that Cas12a-introduced DSBs could be efficiently repaired by either NHEJ or HDR, which therefore facilitates convenient genome editing in *A. mediterranei*.

We first tested dCas9 but found the protein was toxic to U32. As Cas9 has been demonstrated to be toxic in many other species (Jiang et al., 2017; Cho et al., 2018), we here directly used Cas12a to construct the genome editing system in U32 instead of testing the wild type Cas9. As the transformation efficiency of the plasmids containing *Cas12a* was much lower than that of the control plasmid, the *Cas12a* gene might also be harmful to U32 cells. However, once transformants were obtained, both the growth rate and the rifamycin yield of these transformants expressing Cas12a were similar to those of the wild type U32, and Cas12a was therefore employed to develop the genome editing system in U32.

Because of the relatively low transformation and recombination efficiencies in the genus *Amycolatopsis*, it is difficult to construct markerless mutants in this genus. Although an electroporation transformation system has been established in U32 years ago (Ding et al., 2002), the restricted condition for bacterial growth and the complex procedure for preparation of electro-competent cells make it difficult to prepare U32 competent cells of high transformation efficiency. Many factors have been known to affect bacterial transformation efficiency, including the restriction systems. With the CRISPR-Cas12a genome editing system established in this study, these factors can be efficiently modified to improve the U32 transformation efficiency. Furthermore, with the availability of the CRISPR-Cas12a system, precise DSBs can be introduced by the crRNA-guided Cas12a cleavage, then either NHEJ or HDR can be employed to repair the DNA damage, generating desired mutants with no markers left. Furthermore, CRISPR-Cas12a-assisted markerless mutagenesis makes it possible to perform continuous genome editing operations. As there is only one available self-replicating plasmid origin in U32, the original plasmid must be eliminated before a new plasmid can be transformed, expressing a new crRNA to target a new locus. Fortunately, plasmid curing experiments showed that nearly all original plasmids could be eliminated after one or two generations of passage in medium without selective pressure of antibiotics (Supplementary Figure S6). Alternatively, a new plasmid carrying a different antibiotic resistance cassette can be directly transformed and the transformants can be cultured on plates with the new antibiotic. Moreover, the new plasmid may also express a crRNA targeting the original antibiotic resistance cassette to help clear the original plasmid.

Cas12a is so far the most minimalistic of CRISPR systems and can process precursor crRNAs (Fonfara et al., 2016). Based on this characteristic, multiple gene editing can be easily achieved through simply constructing a crRNA array, expressing multiple precursor crRNAs driven under one promoter, which can be further processed by Cas12a to generate multiple mature crRNAs for multiple gene editing or gene regulation *in vivo* (Zetsche et al., 2017; Zhang et al., 2017b). After mutation of the RuvC domain, the DNase-dead Cas12a (namely, ddCas12a) can be employed for gene regulation. Similarly, multiple gene regulation can be achieved with the co-expression of both ddCas12a and a crRNA array (Zhang et al., 2017b). Moreover, with the mutagenesis of the crRNA DR, the ddCas12a binding affinities against mutant crRNAs can be precisely determined and hence the regulatory strength of target genes’ transcription (Wang et al., 2019). Although gene regulation was not tested in this study, one may easily perform transcriptional regulation through simply changing the wild type Cas12a to ddCas12a.

There are vast majority of biosynthetic clusters in the genus *Amycolatopsis*, demonstrating the genus has great potential to produce diverse secondary metabolites (Adamek et al., 2018). Without efficient genome editing tools, heterologous expression is the main way to produce and characterize these metabolites, which could be of low efficiency. As CRISPR-based genome editing has been demonstrated as an efficient approach to discover unique metabolites in *Streptomyces* (Zhang et al., 2017a), the present work will certainly shed light on the development of CRISPR-assisted genome editing systems in other species in *Amycolatopsis* genus and further facilitates the genome mining in this genus.

## Data Availability Statement

All datasets generated for this study are included in the article/Supplementary Material.

## Author Contributions

YZ and XL performed most of the experiments. YZ prepared the draft. JWu drew the schematic maps. JWa and GZ designed the study. JWa revised the manuscript and supervised the whole project.

## Conflict of Interest

The authors declare that the research was conducted in the absence of any commercial or financial relationships that could be construed as a potential conflict of interest.
