# Supplementary material for: CRISPR-Cas12a-Assisted Genome Editing in Amycolatopsis mediterranei
Source: Front Bioeng Biotechnol. 2020 Jun 26;8:698. doi: 10.3389/fbioe.2020.00698 (PMC7332547; doi:10.3389/fbioe.2020.00698)
Supplement: Supplementary file 1 [file Data_Sheet_1.PDF]

Supplementary Data

Supplementary Tables

Table S1. Strains and plasmids used in this study

Strain/Plasmid	Description	Source /reference
**Strains**		
E. coli DH10B	Cloning vector for plasmid construction	This laboratory
A. mediterranei U32	Wild type *A. mediterranei* strain	This laboratory
U32(Cas12a)	U32 strain with pDZL*Cas12a* integrated at its *attB* site	This work
**Plasmids**		
pDZL803	An integrative vector in U32 (*aac3(IV)*; Hyg^r; *oriT*; pUC18-ori; BT1-*int*)	This laboratory
pJV53-*Cpf1*	The *FnCpf1* expression plasmid (*oriM*; *oriE*; Kan^r; Che9c; *FnCpf1*; P_{myc1}tetO promoter)	(Yan et al. 2017)
pDZL*Cas12a*	pDZL803 inserted with the *FnCas12a* (*FnCpf1*) gene amplified from pJV53-*Cpf1*	This work
pCR-HYG	The crRNA expression cassette plasmid (P_{hsp60}; DR; BpmI and HindIII sites; *rrnB* T1 terminator; Hyg^r)	(Yan, Yan, Ren, Zhao, Guo and Sun 2017)
pCR1	Constructed crRNA expression plasmid on the basis of pCR-HYG, with two restriction sites (BpmI and HindIII) replaced to BbsI and AseI (Hyg^r)	This work
pULVK2A	A stably replicable plasmid in U32	This laboratory
pULcrRNA	pULVK2A inserted with the crRNA expression cassette amplified from pCR1	This work
pUL*rifZ*1	*RifZ* guide sequence 1 inserted in pULcrRNA, targeting the T strand (pBR-ori; Apr^r)	This work
pUL*rifZ*2	*rifZ* guide sequence 2 inserted in pULcrRNA, targeting the T strand (pBR-ori; Apr^r)	This work
PUL*rifZ*3	*rifZ* guide sequence 3 inserted in pULcrRNA, targeting the NT strand (pBR-ori; Apr^r)	This work
pUL*glnR*1	*glnR* guide sequence 1 inserted in pULcrRNA, targeting the NT strand (pBR-ori; Apr^r)	This work
pUL*glnR*2	*glnR* guide sequence 2 inserted in pULcrRNA, targeting the NT strand (pBR-ori; Apr^r)	This work
PUL*glnR*3	*glnR* guide sequence 3 inserted in pULcrRNA, targeting the T strand (pBR-ori; Apr^r)	This work
pBCAm	A plasmid contains the apramycin resistance cassette (Apr^r)	This laboratory
pUL*rifZ*1-LAR	pUL*rifZ*1 inserted with a donor containing the upstream and downstream homologous sequences and apramycin resistance cassette (LAR) (pBR-ori; Apr^r)	This work
pUL*rifZ*2-LAR	pUL*rifZ*2 inserted with a donor containing the upstream and	This work

	downstream homologous sequences and apramycin resistance cassette (LAR) (pBR-ori; Apr^r)	
pUL*rifZ*3-LAR	pUL*rifZ*3 inserted with a donor containing the upstream and downstream homologous sequences and apramycin resistance cassette (LAR) (pBR-ori; Apr^r)	This work
pUL*rifZ*1-LR	pUL*rifZ*1 inserted with a markerless donor containing the upstream and downstream homologous sequences (LR) (pBR-ori; Apr^r)	This work
pUL*rifZ*2-LR	pUL*rifZ*2 inserted with a markerless donor containing the upstream and downstream homologous sequences (LR) (pBR-ori; Apr^r)	This work
pUL*rifZ*3-LR	pUL*rifZ*3 inserted with a markerless donor containing the upstream and downstream homologous sequences (LR) (pBR-ori; Apr^r)	This work
pUL*glnR*1-LR	pUL*glnR*1 inserted with a markerless donor containing the upstream and downstream homologous sequences (LR) (pBR-ori; Apr^r)	This work
pUL*glnR*2-LR	pUL*glnR*2 inserted with a markerless donor containing the upstream and downstream homologous sequences (LR) (pBR-ori; Apr^r)	This work
pUL*glnR*3-LR	pUL*glnR*3 inserted with a markerless donor containing the upstream and downstream homologous sequences (LR) (pBR-ori; Apr^r)	This work

Table S2. Oligonucleotides used in this study

**Primer**	**Sequence (5'→3')**	**Description**
pDZL803-apr-F	cagtcgatcatagcacgatc	Construction of plasmid pDZLCas12a
pDZL803-apr-R	gctcatgagcggagaacga	
FnCas12a-F	gatcgtgctatgatcgactgATGTCGATCTACCAAGAGTTC	Amplification of *FnCas12a* gene from pJV53-*Cpf1*
FnCas12a-R	ctcgttctccgctcatgagcTCAATTGTTGCGATTTTGGA	
pCR-HYG - F	actgttgtagatatcgactgccaggcatcaaataaaacgaaaggctcagtcgaaagactgggcctttcgttttatgccatcatggccgcgggctagctt	Replacement of the HindIII and BpmI sites in pCR-HYG to BbsI and AseI
pCR-HYG -R	agaaattatttaaagttcttagacATTAATcactagcatgaagacaaatctacaacagtagaaattatttaaagttcttagacccgttttgcctaa	
hsp60-rrnB-F	gttaccgtgagctgcagcccaagctTAGACGGTGACCACAACGCGC	Amplification of the crRNA expression cassette from plasmid pCR1
hsp60-rrnB-R	tcgcgaggggatcgagcccgggagatctactagTAGCCCGCGGCCATGATGG	
glnRcrRNA1-F	AGATTGGTACTGACTGCAGAGGCC	Cloning of the crRNA protospacer targeting *glnR*1
glnRcrRNA1-R	TAGGCCTCTGCAGTCAGTACCA	

glnRcrRNA2-F<br>glnRcrRNA2-R	AGATCGCGCAGGTCGACGCCGAGC<br>TAGCTCGGCGTCGACCTGCGCG	Cloning of the crRNA protospacer targeting *glnR*2
glnRcrRNA3-F<br>glnRcrRNA3-R	AGATCGCAGGCGCGCGGTGTAGGT<br>TAACCTACACCGCGCGCCTGCG	Cloning of the crRNA protospacer targeting *glnR*3
rifZcrRNA1-F<br>rifZcrRNA1-R	AGATCTGATCAAGGCCCGGGACCT<br>TAAGGTCCCGGGCCTTGATCAG	Cloning of the crRNA protospacer targeting *rifZ*1
rifZcrRNA2-F<br>rifZcrRNA2-R	AGATCGTCGTGCTGGCGCTGGGTG<br>TACACCCAGCGCCAGCACGACG	Cloning of the crRNA protospacer targeting *rifZ*2
rifZcrRNA3-F<br>rifZcrRNA3-R	AGATTGCTCGACGGCGGTCATGCG<br>TACGCATGACCGCCGTCGAGCA	Cloning of the crRNA protospacer targeting *rifZ*3
rifZL-F<br>rifZL-R	tactgagagtgcaccatatgGTGGATCAGGCCGGAGATGATG<br>gcatcagttaccgtgagctgcagTAGCGGGCAAACGGTTAGTAGT	Amplification of the upstream homologous arm of *rifZ*
rifZR-F<br>rifZR-R	ttgacattggggaattcctgcagCGTGGAAGGTGTTCTCGTAGGC<br>ggtatttcacaccgcatatgGTGCCGTTGATGACGAAGGTGC	Amplification of the downstream homologous arm of *rifZ*
glnRL-F<br>glnRL-R	tactgagagtgcaccatatgTTCATACCTTGGGACGATTC<br>GGTCGGTGCTAAGTGAGAAT	Amplification of the upstream homologous arm of *glnR*
glnRR-F<br>glnRR-R	attctcacttagcaccgaccGGCCGAATGACTGCGTAGTA<br>ggtatttcacaccgcatatgACCGCTCGGGTTGTAGTTGA	Amplification of the downstream homologous arm of *glnR*
rifZ-KO-F<br>rifZ-KO-R	AGCAGCAGGTTGGCGATGTT<br>GTCCTTGTCGCTCGTCTTGT	PCR verification of *rifZ* mutants using primers outside of the homologous arms, and the amplicons were used for Sanger sequencing
glnR-KO-F<br>glnR-KO-R	CTCTTCCTTGGTTGTTCATC<br>GGGTGTTGCTGGCGTAGATG	PCR verification of *glnR* mutants using primers outside of the homologous arms, and the amplicons were used for Sanger sequencing
rifZ+P-F<br>rifZ+P-R	ACCGTGAACATCGGCAGACC<br>AactagtCTGGGGCAGCCGGACCTCC	PCR verification of *rifZ* mutants using primers inside of the homologous arms
U32-glnRP-F<br>GlnR-MV-R	CCTGGGTGAGGAACGGGAA<br>GCGACGGCTGTGCGAGACG	PCR verification of *glnR* mutants using primers inside of the homologous arms

Table S3. crRNA guide sequences used in this study

crRNA	Guide sequences (5'-3')	PAM	Target strand
rifZ-1	CTGATCAAGGCCCGGGACCT	TTG	T
rifZ-2	CGTCGTGCTGGCGCTGGGTG	TTA	T
rifZ-3	TGCTCGACGGCGGTCATGCG	TTG	NT
glnR-1	TGGTACTGACTGCAGAGGCC	TTC	NT
glnR-2	CGCGCAGGTCGACGCCGAGC	TTC	NT
glnR-3	CGCAGGCGCGCGGTGTAGGT	TTG	T

Table S4. BLASTP results of candidate DNA ligase D in U32 with the amino acid sequences of MSMEG_5570 as the query sequences

genes	alignment length	mismatches	E value	Per. Ident
AMED_5275	350	158	1.31E-28	22.286
AMED_4189	177	79	1.17E-21	24.294
AMED_3255	266	101	1.33E-13	29.323
AMED_4197	156	52	9.24E-13	34.615
AMED_3255	232	100	5.08E-12	19.397
AMED_5204	233	107	1.54E-09	20.172
AMED_4148	185	91	1.95E-07	15.135
AMED_8064	186	80	1.53E-06	24.194
AMED_4489	107	54	6.47E-06	26.168

Table S5. BLASTP results of candidate DNA end-binding protein Ku protein in U32 with the amino acid sequences of MSMEG_5580 as the query sequences

genes	alignment length	mismatches	E value	Per. Ident
AMED_5266	285	140	8.54E-31	21.404
AMED_5205	298	148	8.81E-23	18.792
AMED_1951	285	160	7.70E-18	18.596

Supplementary Figures

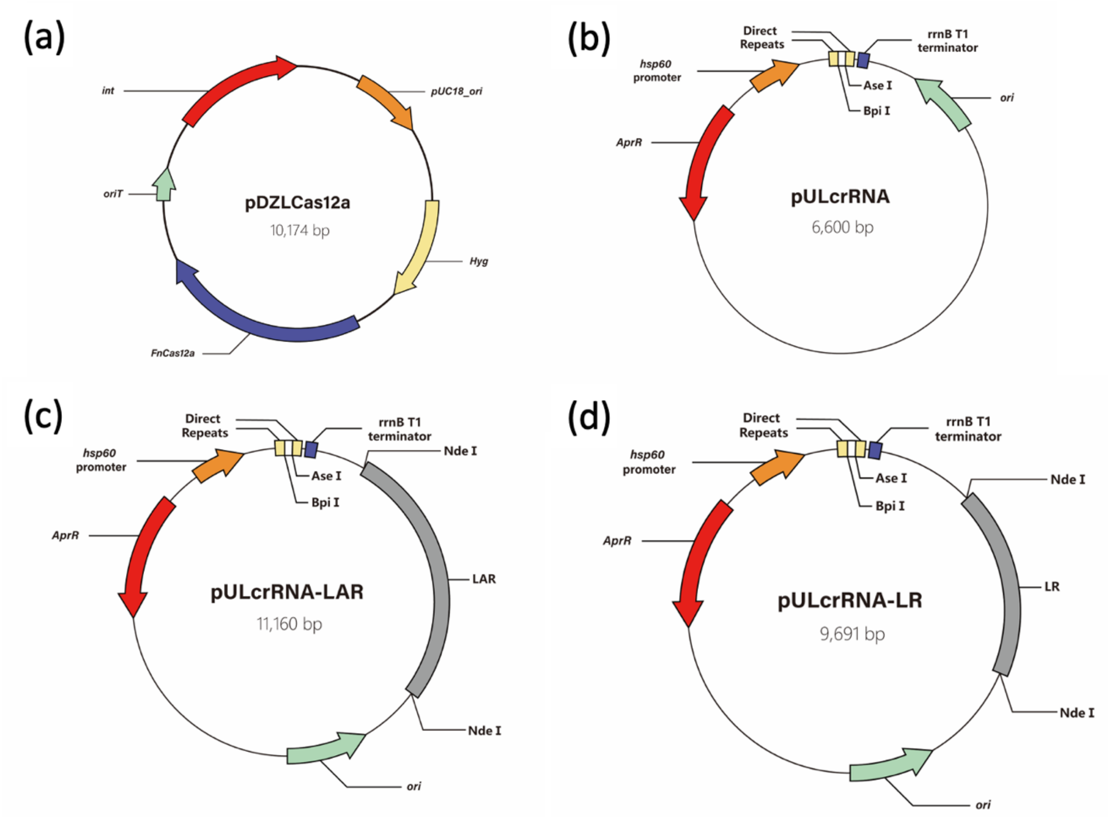

Figure S1. Schematic of the CRISPR-FnCas12a-assisted two-plasmid gene editing system. (a) Plasmid pDZLCas12a was first transformed into U32 and integrated into the chromosome to allow for constitutive expression of FnCas12a. (b) The crRNA expression plasmid with the Direct Repeats for cloning the guide sequences into the BpiI and AseI sites. (c) Schematic map of the crRNA expression plasmid containing the LAR recombination sequences. (d) Schematic map of the crRNA expression plasmid containing the LR recombination sequences.

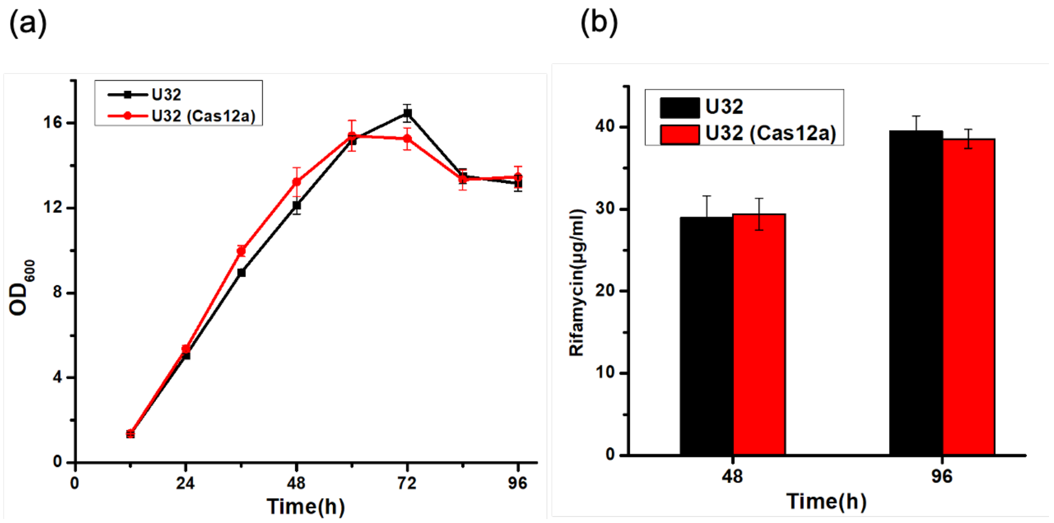

Figure S2. Influence of Cas12a on bacterial growth and rifamycin yield. Both U32 and U32(Cas12a) that harbored the *FnCas12a* gene were cultured in liquid Bennet medium. Their growth curves were determined by the measurement of the OD600 values (a), and their rifamycin yield was measured with the spectrophotometric method (b).

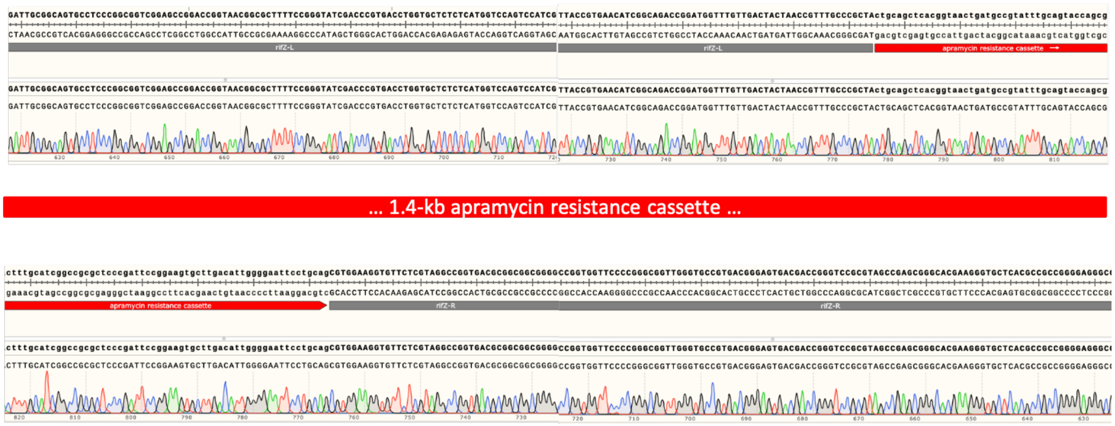

Figure S3. Sequencing results encompassing the rifZ-L, apramycin resistance cassette and rifZ-R fragments. The sequencing results were viewed and aligned with the SnapGene software.

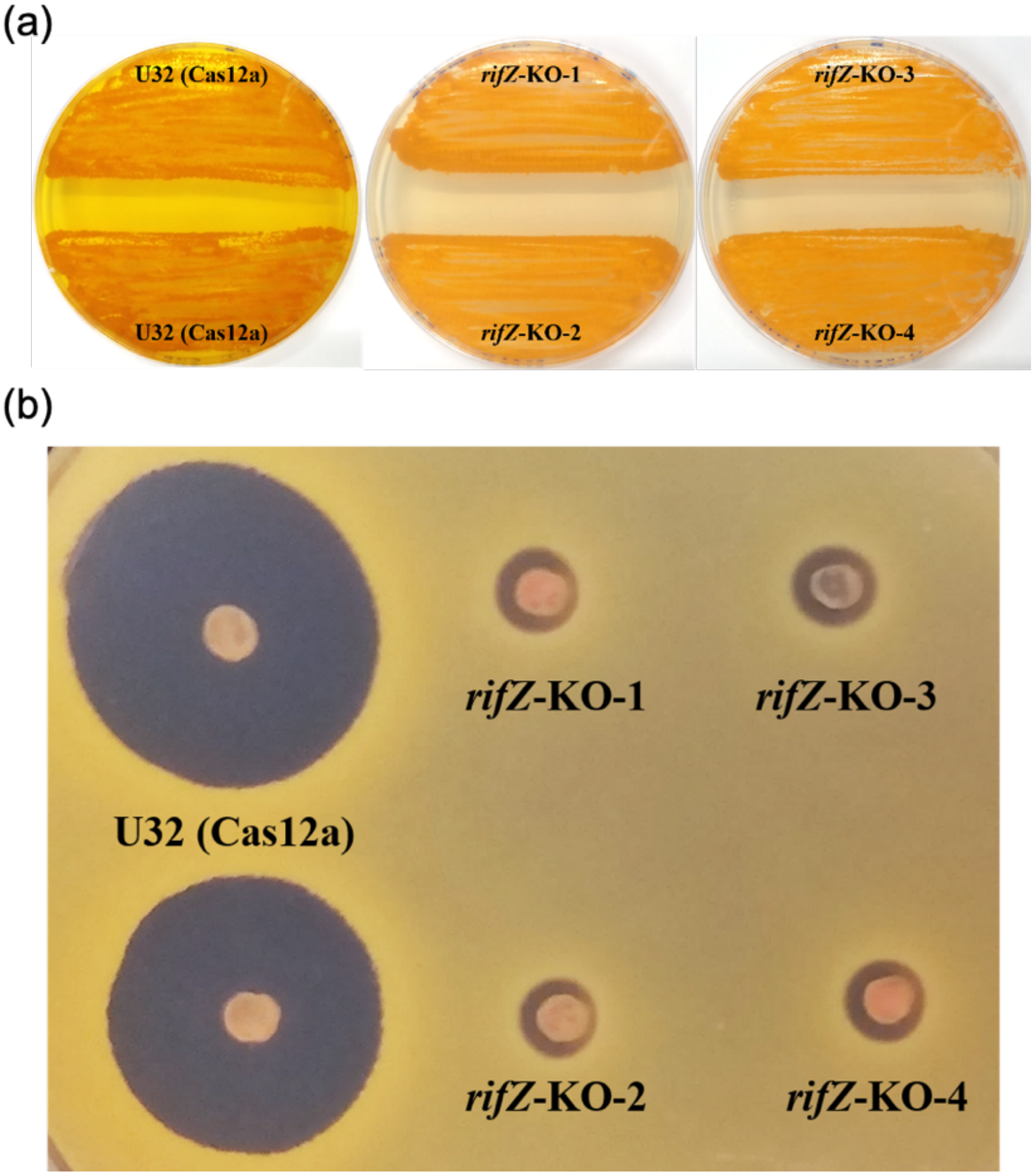

Figure S4. Growth phenotypes and the rifamycin yields of rifZ-KO mutants. Both the wild type U32 and the *rifZ*-KO mutants were grown on Bennet agar for 5 days before the observation of the pigment production (a) and rifamycin SV production by bactericidal test with *Sarcina lutea* as the indicate strain (b).

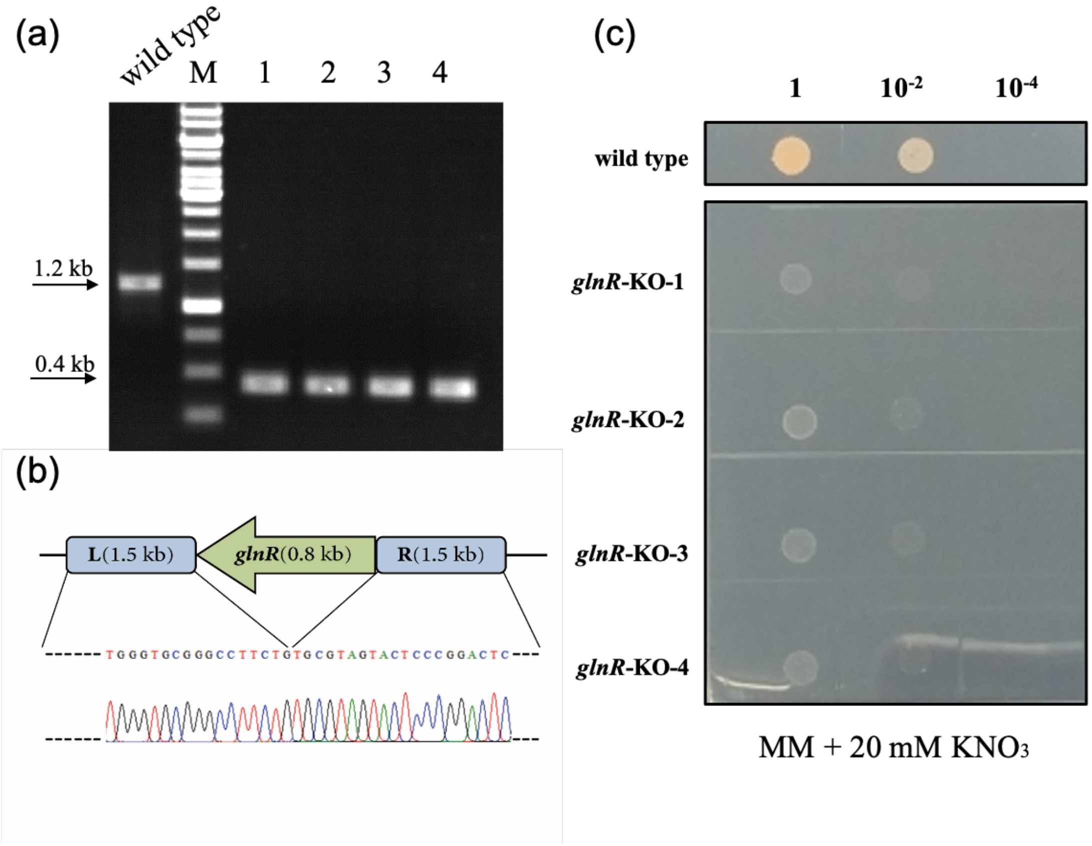

Figure S5. Markerless deletion of *glnR* gene with the Cas12a and HDR systems in U32. (a) Verification of *glnR* transformants by primers of U32-glnRP-F and GlnR-MV-R. The 0.4-kb amplicon from the in-frame deletion mutants was obviously smaller than that from the wild type, which was about 1.2 kb in length. M, GeneRuler 1 kb DNA Ladder (Thermo Scientific). Lanes 1-4 represented *glnR*-KO-1 to *glnR*-KO-4, respectively. (b) Confirmation of the *glnR* null mutants *via* Sanger sequencing. The sequencing data showed that the *glnR* gene was precisely in-frame deleted in all transformants, and only the result from mutant *glnR*-KO-1 in **Fig. S5a** was presented. (c) Growth phenotype analysis of *glnR* null mutants on minimal medium supplemented with nitrate as the sole nitrogen source. Serially diluted liquid culture (1×, 10^2× and 10^4×) was spotted on plates and cultured at 30 °C for 5 days, and all mutants failed to grow on minimal medium.

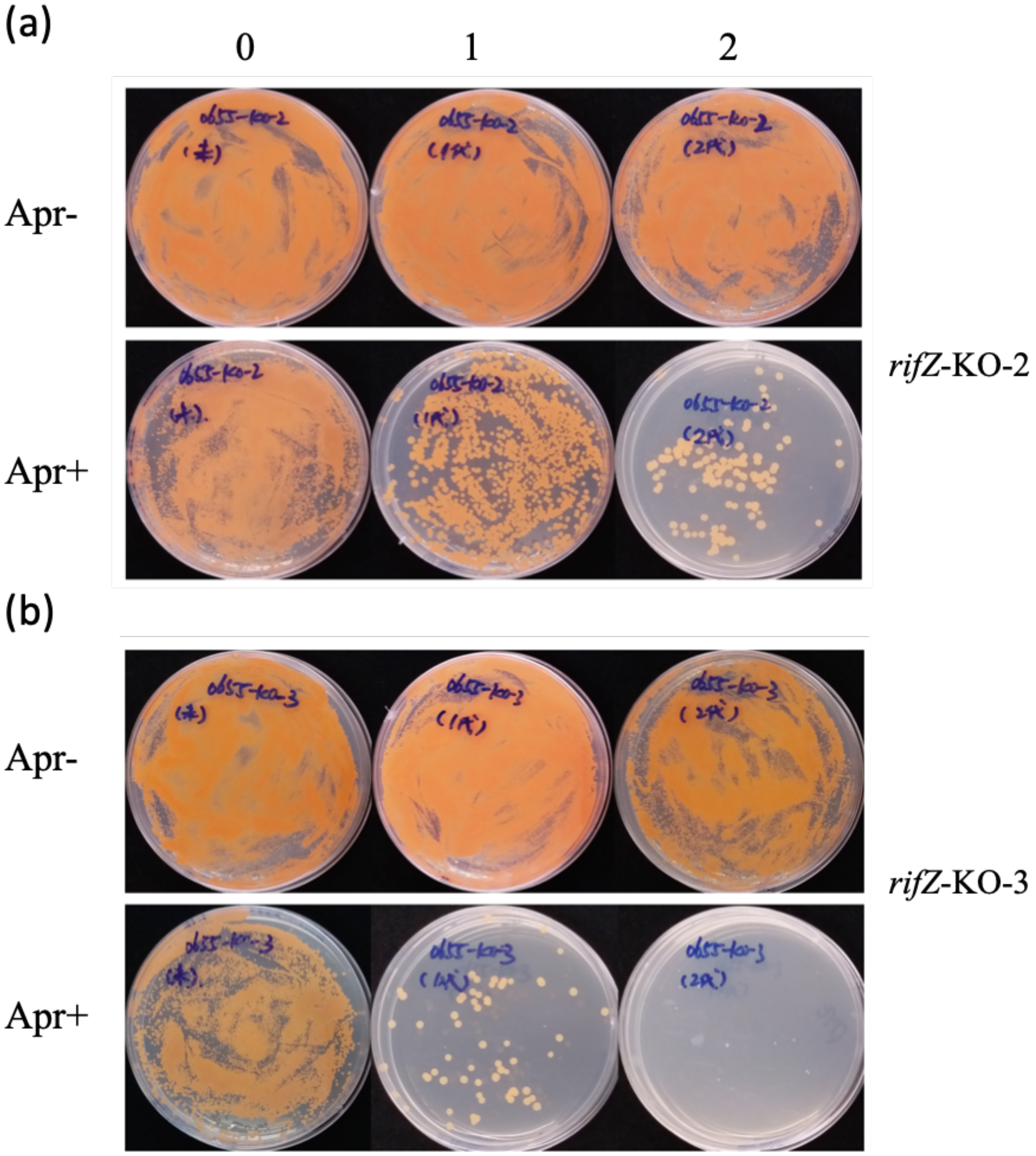

Figure S6. Elimination of self-replicable plasmids in *rifZ*-KO-2 and *rifZ*-KO-3. Strains *rifZ*-KO-2 (a) and *rifZ*-KO-3 (b) were cultured in liquid Bennet medium without antibiotic supplementation for 0, 1 and 2 generations (48 h/generation), and diluted culture was then spread on Bennet plate with or without apramycin supplementation to check the elimination of the crRNA expression plasmids in the strains.

References

Yan MY, Yan HQ, Ren GX, Zhao JP, Guo XP, Sun YC. 2017. CRISPR-Cas12a-Assisted Recombineering in Bacteria. Applied and environmental microbiology. Sep 1;83.
